# Simplifying microbial electrosynthesis reactor design

**DOI:** 10.3389/fmicb.2015.00468

**Published:** 2015-05-15

**Authors:** Cloelle G. S. Giddings, Kelly P. Nevin, Trevor Woodward, Derek R. Lovley, Caitlyn S. Butler

**Affiliations:** ^1^Department of Civil and Environmental Engineering, University of Massachusetts at AmherstAmherst, MA, USA; ^2^Department of Microbiology, University of Massachusetts at AmherstAmherst, MA, USA

**Keywords:** microbial electrosynthesis, bioelectrochemical system, *Sporomusa ovata*, renewable energy storage, artificial photosynthesis, CO_2_ sequestration

## Abstract

Microbial electrosynthesis, an artificial form of photosynthesis, can efficiently convert carbon dioxide into organic commodities; however, this process has only previously been demonstrated in reactors that have features likely to be a barrier to scale-up. Therefore, the possibility of simplifying reactor design by both eliminating potentiostatic control of the cathode and removing the membrane separating the anode and cathode was investigated with biofilms of *Sporomusa ovata*. *S. ovata* reduces carbon dioxide to acetate and acts as the microbial catalyst for plain graphite stick cathodes as the electron donor. In traditional ‘H-cell’ reactors, where the anode and cathode chambers were separated with a proton-selective membrane, the rates and columbic efficiencies of microbial electrosynthesis remained high when electron delivery at the cathode was powered with a direct current power source rather than with a potentiostat-poised cathode utilized in previous studies. A membrane-less reactor with a direct-current power source with the cathode and anode positioned to avoid oxygen exposure at the cathode, retained high rates of acetate production as well as high columbic and energetic efficiencies. The finding that microbial electrosynthesis is feasible without a membrane separating the anode from the cathode, coupled with a direct current power source supplying the energy for electron delivery, is expected to greatly simplify future reactor design and lower construction costs.

## Introduction

Microbial electrosynthesis shows promise as a strategy for the production of transportation fuels and other organic commodities from carbon dioxide with renewable electricity as the energy source (Leang et al., 2012; LaBelle et al., 2014; Rosenbaum and Henrich, 2014). In microbial electrosynthesis, microorganisms accept electrons from a cathode for the reduction of carbon dioxide to organic compounds that are excreted from the cell (Nevin et al., 2010, 2011; Lovley and Nevin, 2011, 2013; Marshall et al., 2013b; Rosenbaum and Henrich, 2014). When electrical energy is derived from solar technologies, microbial electrosynthesis is an artificial form of photosynthesis that has much higher potential energetic efficiencies and avoids many of the environmental and societal concerns associated with large-scale biomass cultivation and conversion to fuels (Nevin et al., 2011; Pant et al., 2011; Desloover et al., 2012; Lovley and Nevin, 2013).

One challenge for commercialization of microbial electrosynthesis is that pure cultures will likely be required in order to directly produce high-value fuels or other organic commodities. Initiating microbial electrosynthesis with mixed cultures yields systems that produce primarily acetate (Marshall et al., 2012, 2013a,b) and likely can be attributed to the metabolism of acetogenic microorganisms. Pure culture studies have demonstrated that a diversity of acetogens can accept electrons from negatively poised electrodes for the reduction of carbon dioxide to acetate with high columbic efficiencies (Nevin et al., 2010, 2011). In order to produce higher value products via electrosynthesis, it will be necessary to genetically modify acetogens to divert carbon and electron flow to the generation of other products (Lovley and Nevin, 2013). The development of a genetic tool box for the acetogen *Clostridium ljungdahlii*, and the use of these tools to redirect carbon and electron flow, suggest that this necessary step in the development of commercially competitive microbial electrosynthesis can be met (Leang et al., 2012; Tremblay et al., 2013; Banerjee et al., 2014; Ueki et al., 2014).

Another major challenge is the design of a robust reactor for microbial electrosynthesis. To date, microbial electrosynthesis has relied on cathodes that have a potential that is carefully controlled with a potentiostat. Potentiostat control was employed in initial studies (Lovley and Nevin, 2013; Zhang et al., 2013) to maintain cathode potentials that would prevent production of H_2_, and to avoid cathode potential fluctuations that might damage cells. Potentiostat control over large electrode systems is impractical because it is energy intensive to maintain a fixed potential and the potentiostat has limited control in large-scale systems (Rosenbaum et al., 2011).

Another concern is that the anode chamber and cathode chamber in previous microbial electrosynthesis reactors were separated with a membrane designed to permit ion flux between the chambers, while restricting oxygen diffusion. Oxygen produced at the anode and reaching the cathode will abiotically consume electrons, reducing reactor efficiency. Furthermore, the acetogenic microorganisms that serve as the catalysts for microbial electrosynthesis are among the most oxygen-sensitive of microorganisms. Finally, membranes add substantial cost, and designing large-scale reactors with two chambers separated by a membrane will be challenging.

The purpose of the study described here was to determine if microbial electrosynthesis reactors could be simplified by removing potentiostat control of the cathode and reconfiguring the anode and cathode to make it possible to avoid a separator membrane. The results suggest that microbial electrosynthesis reactors can be simplified in this manner while maintaining high energetic efficiencies.

## Materials and Methods

### Culturing Techniques in Microbial Electrosynthesis Reactors

The acetogen *Sporomusa ovata* was grown on electrons from an electrode in the cathode chamber of an H-cell reactor. Unmodified or control reactors contained graphite stick anodes and cathodes (65 cm^2^; Mersen, Greenville, MI, USA) suspended in two chambers, each containing 200 ml of modified DSMZ 311 defined growth medium (betaine, casitone, resazurin, and yeast extract omitted) media, that were separated with a Nafion 117 cation-exchange membrane (Electrolytica, Amherst, NY, USA), which had an effective working area of 6.25 cm^2^. Reactor modifications are described below.

*Sporomusa ovata* for all experiments was initially grown in 50 mL of modified DSMZ 311 defined growth medium (yeast extract omitted) with hydrogen as the electron donor [H_2_–CO_2_ (80:20)] in 156 mL serum bottles, as described previously (Nevin et al., 2011). After the reactors were inoculated (40% v/v, unless otherwise stated; in same medium), the culture was bubbled with a gas mixture containing 7% hydrogen [N_2_–CO_2_–H_2_ (80:13:7)] to help establish a biofilm on the cathode. Fifty percent of the medium was replaced once acetate concentrations were above 10 mM.

After three media-exchanges in each reactor configuration, the culture was switched to a different gas mixture [N_2_–CO_2_ (80:20)] that contained no hydrogen. After 2 days, the system was changed from a batch to flow-through process, where fresh medium was continuously added at 0.02 mL/min. A direct current power source provided a potential difference between the anode and cathode. Cathode potential, current, and total applied voltage was measured using a digital voltmeter. The total voltage applied was between 1.9 to 5 V, depending on the reactor system. Approximately 0.2 mL of liquid was regularly sampled for acetate measurement and the headspace of the reactors were sampled periodically to test for hydrogen production. All reactors were incubated at 25∘C.

### Reactor Schemes

#### Microbial Electrosynthesis Reactors

Unmodified reactors were run as controls to compare to the reactors studied with poised cathode potentials and were constructed as described in Section “Culturing Techniques in Microbial Electrosynthesis Reactors.” Additionally, two H-cells were run with an increased applied voltage over the system to boost current density and to encourage acetate production (**Table 1**).

**Table 1:** Summary of reactor designs and experimental program.

Configuration	Objective	Voltage delivered (V)	Cathode potential [V vs. standard hydrogen electrode (SHE)]
H-cells	H-cells poised at different cell potentials	3.0, 3.5, 5.0	-0.65, -0.71, -0.74
Abiotic	Measure hydrogen production in the absence of microbes	3.5	-0.79
Membrane-less	Decrease resistance of the reactors to increase energy efficiency	1.9 to 2.5	-0.70 to -0.81

#### Abiotic Reactors

Abiotic reactors were constructed identically to the unmodified H-cell reactors. A voltage was applied, and the working side of the reactor was sampled daily for hydrogen to explore hydrogen production in the absence of microorganisms (**Table 1**).

#### Membrane-Less Reactors

**FIGURE 1:**
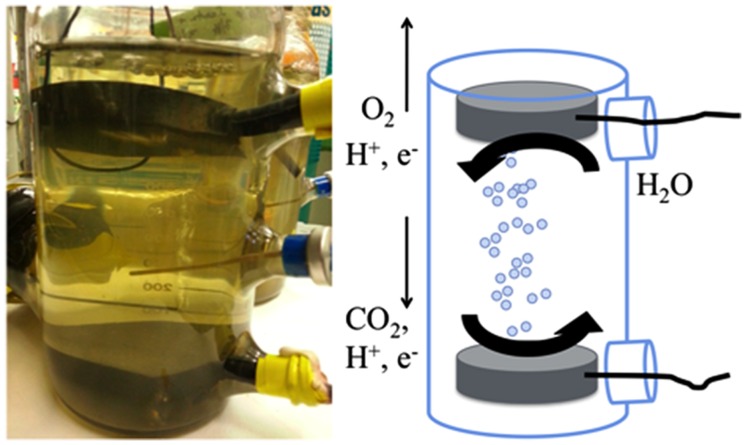
**Configuration of membrane-less reactors**.

To decrease the internal resistance of our reactors, we constructed a reactor without a proton exchange membrane. The reactor was housed in a modified 1-L pyrex glass media bottle (Corning) that had three 20 mm serum ports (**Figure 1**). The cathode and anode were circular graphite disks, each with a surface area of 1.2 × 10^-2^ m^2^ (diameter of 7.6 cm, height of 1.3 cm). A blunt-end cannula for gassing was placed between the electrodes to help keep oxygen generated at the anode from diffusing down to the cathode. Initially, the applied voltage to the reactor was 2.5 V, however, this was lowered over the course of the experiment to limit hydrogen production (**Table 1**). *S. ovata* culturing for the membrane-less reactors was as described above, but the initial inoculation level was lower (∼15% v/v) as there was limited culture for these larger volume reactors because we maintained pure culture for inoculum in small volumes (50 mL). During operation, fresh media was supplied at a flow rate of 0.01 mL/min. An abiotic control of the membrane-less reactor was performed but a stable voltage could not be sustained and no acetate was produced.

### Analytical Methods

Acetate was measured via high performance liquid chromatography (HPLC). Organic acids were separated on an Aminex NPX-87H column with 8 mM H_2_SO_4_ as the eluent and detected at 210 nm. Hydrogen was measured by running headspace samples on an Agilent 6890 gas chromatograph [Carbonex-1010 PLOT capillary column (Supelco); He carrier gas at 12 ml min^-1^ total flow; oven temperature 40∘C; thermal-conductivity detector (TCD) temperature 225∘C]. Aqueous phase hydrogen concentrations were assumed to be negligible due to stripping by the continuous gassing and because of the low solubility of H_2_ gas. Cathode potentials were measured *in situ* vs. an Ag/AgCl reference electrode [+0.197 V vs. standard hydrogen electrode (SHE)]. All potentials have been adjusted and reported versus SHE.

### Electron Recovery and Reactor Efficiency

The columbic efficiency (CE) was found by comparing the number of electrons appearing in acetate, based on Eq. 1, to electrons consumed as current (Eq. 2).

(1) $$2{CO}_{2}+8e^{-}+8H^{+}+2H_{2}O\to {CH}_{3}COOH+2O_{2}$$

(2) $$CE\left(\%\right)=\frac{nFm_{acetate}}{\int_{0}^{t}I\mathrm{d}t}\times 100\%\;$$

Where, *n* is the moles of electrons required to reduce carbon dioxide to one mole of acetate (i.e., eight electrons), *F* is Faraday’s constant (96485 C mol^-1^), m is the mass of acetate produced from *t_0_* to *t*. *I* is the current delivered to the reactor, which when integrated over time *t_0_* to *t* yields the total Coulombs transferred to the system to produce the measured acetate.

The reactor efficiency, η_ME_, was found by comparing the energy content of the product (per mol of acetate) to the energy delivered to the reactor (Eq. 3),

(3) $$\eta ME=\frac{E_{out}}{E_{in}}\times 100\%=\frac{E_{acetate}}{E_{potential}}\times 100\%\;$$

where *E_acetate_* is the theoretical energy produced from acetate based on oxidation with oxygen as an electron acceptor (848 kJ mol^-1^), and *E_potential_* is the sum of Gibb’s energy of formation of acetate and the energy required to apply the voltage to the system based on microbial kinetic parameters which are assumed to be similar to hydrogen-oxidizing acetogenensis (Rittmann and McCarty, 2001), and varied depending on the voltage delivered. For example, at an applied voltage of 1 V, the total *E_potential_* is 991 kJ mol^-1^.

## Results and Discussion

### Delivery of Electrons with a DC Power Source

Previous studies of electrosynthesis have poised the cathode at a fixed potential with a potentiostat. However, such a poised system would be difficult to control at a large scale. Therefore, the possibility of delivering electrons to the cathode with a DC power source was evaluated in ‘H-cell’ reactors similar to those used in previous studies with potentiostat-poised cathodes (Nevin et al., 2010, 2011).

The H-cell reactor required that a voltage of 3.0 V or higher be applied in order to maintain a stable cathode voltage. Applied voltages less than 3.0 V yielded a more positive cathode potential that was closer to the -0.4 V of previous studies (Nevin et al., 2010, 2011), but at applied voltages less than 3.0 V made it difficult to maintain a stable cathode potential.

With applied voltages of 3.0 or 3.5 V (1), *S. ovata* produced 0.45 and 0.54 mmoles of acetate per day, respectively (**Table 2**). These rates are nearly threefold higher than the rates previously reported in the same reactors with the cathodes poised at -0.4 V with a potentiostat (Nevin et al., 2010, 2011). This improvement in performance may be attributed in part to the fact that the cathode potentials in the DC power source systems (**Table 2**) were lower than the -0.4 V potential of the potentiostat-poised system (Nevin et al., 2010).

**Table 2:** Performance of reactors varying voltage delivery.

Configuration^∗^	Voltage delivered (V)	Current density (A/m^2^)	Power (mW)	Cathode potential (V vs. SHE)	Acetate production			CE (%)	ηME (%)
					(mmole/day)	(mmole/day/m^2^ cathode)	(g/kWh)		
H-cell (1)	3.0	0.71 ± 0.06	13.8 ± 1.1	-0.65	0.45 ± 0.17	69 ± 26	81.4 ± 31.5	82 ± 19%	33%
H-cell (2)	3.5	0.87 ± 0.08	19.8 ± 1.8	-0.73	0.54 ± 0.08	83 ± 12	68.3 ± 11.9	83 ± 11%	29%
H-cell (3)	5.0	1.70 ± 0.19	55.2 ± 6.2	-0.74	1.07 ± 0.29	164 ± 45	48.5 ± 14.2	89 ± 12%	21%
Abiotic H-cell	3.5	0.77 ± 0.05	15.1 ± 3.3	-0.79	–	–	–	–	–
Membrane-less	1.9	0.17 ± 0.04	3.9 ± 0.6	-0.66	0.34 ± 0.05	28 ± 4	218.6 ± 64.3	105 ± 5%	50%
	2.2	0.25 ± 0.06	6.9 ± 1.9	-0.68	0.45 ± 0.02	38 ± 2	167.4 ± 37.1	79 ± 6%	44%
	2.5	0.46 ± 0.03	14.4 ± 0.7	-0.81	0.43 ± 0.14	36 ± 12	77.6 ± 25.6	54 ± 10%	39%

*^∗^All reactors were run in duplicate*.

**FIGURE 2:**
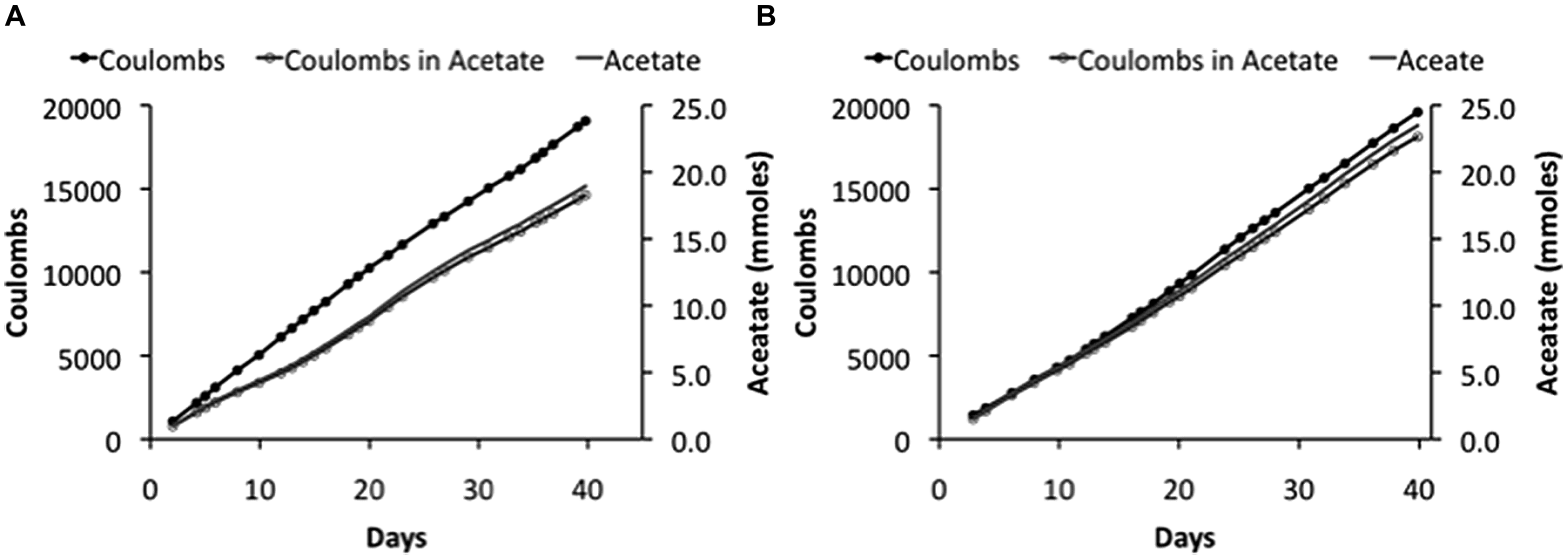
**Coulombs applied and recovered as acetate and acetate production in duplicate (A) and (B) “H-cell” reactors at an applied voltage of 3.5V**.

In fact, the cathode potentials (-0.65 to -0.73 V) were theoretically low enough to produce H_2_. However, no H_2_ was detected during microbial electrosynthesis with *S. ovata* biofilms. In contrast, in abiotic controls at an applied voltage of 3.5 V, H_2_ concentrations measured in the headspace ranged from 200 to 800 ppmv over the 2 weeks trial and the current density was only slightly less than the current density with *S. ovata* biofilms (**Figure 2**). Therefore, it is possible that *S. ovata* was using H_2_ abiotically produced at the cathode as an electron donor rather than the direct cathode to microbe electron transfer that has been experimentally demonstrated in previous studies with *Geobacter* species (Gregory et al., 2004) and inferred in previous studies of microbial electrosynthesis with acetogenic bacteria (Nevin et al., 2010, 2011). However, the recovery of electrons consumed in acetate produced with the DC power source systems (**Table 2**) was comparable with the previously reported (Nevin et al., 2010) recovery of 86 ± 21% in the potentiostat-poised system with more positive cathode potentials. Therefore, even if H_2_ was the ultimate electron donor for electrosynthesis in the DC power source systems, this was of little practical consequence as the *S. ovata* biofilms were highly effective in scavenging H_2_ before it was lost to the gas stream passing through the reactor.

**FIGURE 3:**
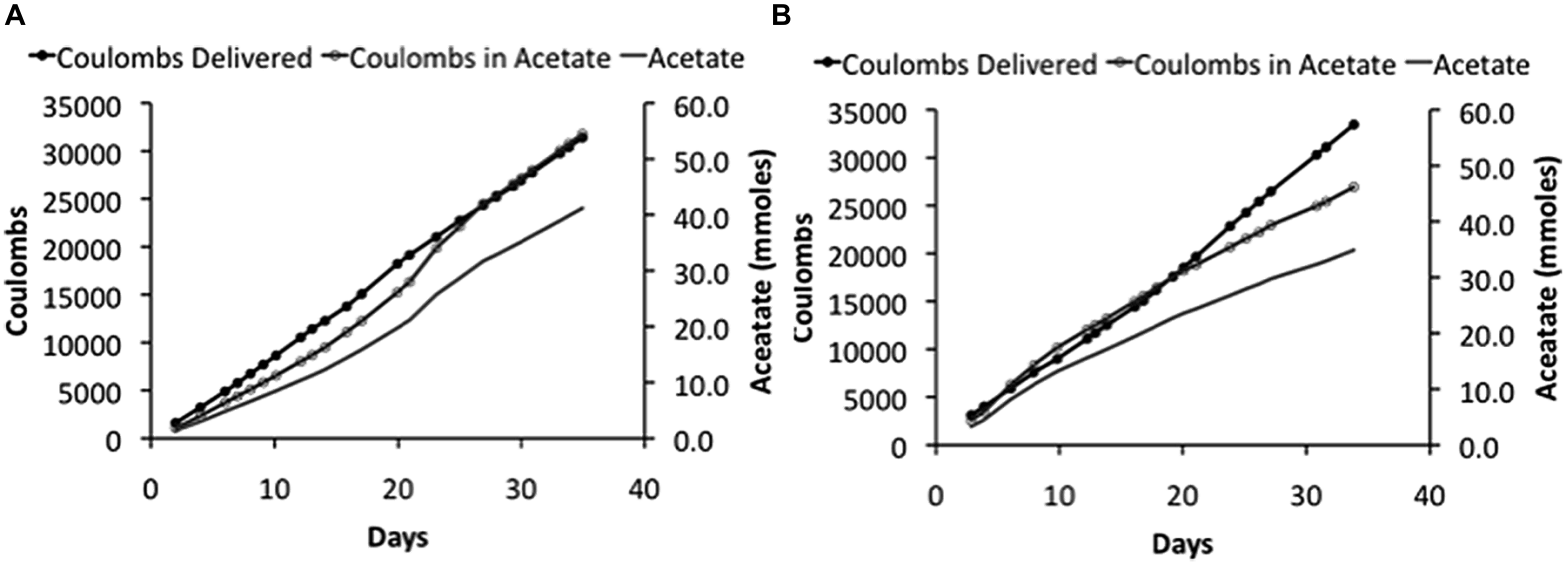
**Coulombs applied and recovered as acetate and acetate -47pcproduction in duplicate (A) and (B) of “H-cell” reactors with an applied voltage of 5 V**.

In order to determine if even higher applied voltages would be effective in yielding greater rates of acetate production, microbial electrosynthesis in an H-cell reactor with an applied voltage of 5.0 V was evaluated. As with the lower applied voltages, there was a steady production of acetate over time (**Figure 3**) with rates of acetate production that were approximately double those obtained with an applied voltage of 3.5 V (**Table 2**). Recovery of electrons consumed in acetate was slightly better than with the lower voltages, demonstrating effective cathode-to-microbe electron transfer whether or not H_2_ was an intermediary electron carrier.

However, the reactor efficiency, which is the ratio of the energy delivered to the reactor to the energy content of the product (per mol of acetate), decreased as the applied voltage was increased (**Table 2**), reflecting the greater power requirement needed to run reactors at higher voltage. For example, the power to provide 5 V was almost three times higher than for the reactors at 3.0 V. Running reactors at higher voltage may be acceptable if there is an excess of electricity to be stored, such as with intermittent renewable energy sources. Otherwise, more conservative voltage schemes might be desirable.

### Membrane-Less Reactor

In order to further simply microbial electrosynthesis reactor design, the possibility of designing a reactor without a membrane separating the anode and cathode was considered. The primary reason for including a membrane in previous microbial electrosynthesis reactor design was to prevent oxygen produced at the anode from coming into contact with the cathode biofilm of *S. ovata*, which is a strict anaerobe. In order to diminish the possibility of oxygen flux to the cathode, the anode was placed at the top of the reactor where the gas flushing through the reactor would remove oxygen produced, reducing downward flux of oxygen toward the cathode (**Figure 1**).

**FIGURE 4:**
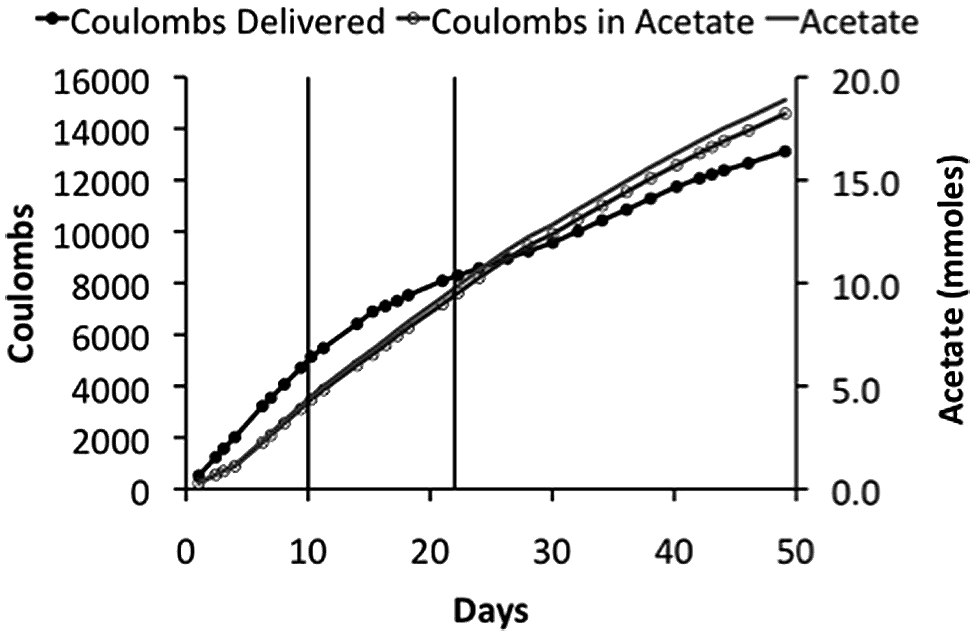
**Coulombs applied and recovered as acetate and acetate production in a membrane-less reactor**. The initial applied voltage was 2.5 V, which was reduced to 2.2 V on day 10, and then to 1.9 V on day 22. These changes in applied voltages are indicated on plot by the vertical lines.

*Sporomusa ovata* thrived in the membrane-less reactor, steadily producing acetate over time (**Figure 4**). However, at the initial applied voltage of 2.5 V, 200–400 ppm H_2_ was detected in the eﬄuent gas, indicating that *S. ovata* was not effectively removing all of the H_2_ produced at the cathode. This loss of H_2_ resulted in a low recovery of electrons in acetate (∼50%), which is unlikely to be cost effective. When the applied voltage was lowered to 2.2 V concentrations of H_2_ were lower (around 150 ppm), but with a comparable acetate production rate, yielding a better electron recovery in acetate (**Table 2**). Further lowering the applied voltage to 1.9 V eliminated detectable H_2_ and further increased the efficiency of electron recovery in acetate (**Table 2**). Furthermore, this high CE was accomplished with a very good recovery of input energy in product (**Table 2**).

## Implications

These results demonstrate that microbial electrosynthesis can be powered with DC power sources at high columbic efficiencies with good recovery of electrical energy supplied in organic product with a membrane-less reactor design. DC power sources are much simpler and inexpensive than poising cathodes with potentiostat-controlled systems, which are unlikely to function effectively with the large cathode surfaces that will be required in commercial-scale reactors. The ability to effectively shield microbial catalyst and the cathode from oxygen produced at the anode in the membrane-less system described here eliminates the substantial costs of effective membranes, and the technical difficulties of designing large reactors with membranes.

The simplification of microbial electrosynthesis with improved energetic efficiency with membrane-less reactors is expected to provide a foundation for the design of larger-scale reactors. Next-generation reactor designs should focus on maximizing cathode surface area with cathode materials optimized to support maximal rates of electrosynthesis (Zhang et al., 2013).

## Conflict of Interest Statement

The authors declare that the research was conducted in the absence of any commercial or financial relationships that could be construed as a potential conflict of interest.

## References

[B1] Banerjee A. Leang C. Ueki T. Nevin K. P. Lovley D. R. (2014). Lactose-inducible system for metabolic engineering of *Clostridium ljungdahlii*. *Appl. Environ. Microbiol.* 80 2410–2416 10.1128/AEM.03666-13 24509933 PMC3993169

[B2] Desloover J. Arends J. B. A. Hennebel T. Rabaey K. (2012). Operational and technical considerations for microbial electrosynthesis. *Biochem. Soc. Trans.* 40 1233–1238 10.1042/BST20120111 23176460

[B3] Gregory K. B. Bond D. R. Lovley D. R. (2004). Graphite electrodes as electron donors for anaerobic respiration. *Environ. Microbiol.* 6 596–604 10.1111/j.1462-2920.2004.00593.x 15142248

[B4] LaBelle E. V. Marshall C. W. Gilbert J. A. May H. D. (2014). Influence of acidic pH on hydrogen and acetate production by an electrosynthetic microbiome. *PLoS ONE* 9:e109935 10.1371/journal.pone.0109935 PMC4198145 25333313

[B5] Leang C. Ueki T. Nevin K. P. Lovley D. R. (2012). A genetic system for *Clostridium ljungdahlii*: a chassis for autotrophic production of biocommodities and a model homoacetogen. *Appl. Environ. Microbiol.* 79 1102–1109 10.1128/aem.02891-12 23204413 PMC3568603

[B6] Lovley D. R. Nevin K. P. (2011). A shift in the current: new applications and concepts for microbe-electrode electron exchange. *Curr. Opin. Biotechnol.* 22 441–448 10.1016/j.copbio.2011.01.009 21333524

[B7] Lovley D. R. Nevin K. P. (2013). Electrobiocommodities: powering microbial production of fuels and commodity chemicals from carbon dioxide with electricity. *Curr. Opin. Biotechnol.* 24 385–390 10.1016/j.copbio.2013.02.012 23465755

[B8] Marshall C. W. LaBelle E. V. May H. D. (2013a). Production of fuels and chemicals from waste by microbiomes. *Curr. Opin. Biotechnol.* 24 391–397 10.1016/j.copbio.2013.03.016. 23587964

[B9] Marshall C. W. Ross D. E. Fichot E. B. Norman R. S. May H. D. (2013b). Long-term operation of microbial electrosynthesis systems improves acetate production by autotrophic microbiomes. *Environ. Sci. Technol.* 47 6023–6029 10.1021/es400341b 23676111

[B10] Marshall C. W. Ross D. E. Fichot E. B. Norman R. S. May H. D. (2012). Electrosynthesis of commodity chemicals by an autotrophic microbial community. *Appl. Environ. Microbiol.* 78 8412–8420 10.1128/aem.02401-12 23001672 PMC3497389

[B11] Nevin K. P. Hensley S. A. Franks A. E. Summers Z. M. Ou J. Woodard T. L. (2011). Electrosynthesis of organic compounds from carbon dioxide is catalyzed by a diversity of acetogenic microorganisms. *Appl. Environ. Microbiol.* 77 2882–2886 10.1128/AEM.02642-10 21378039 PMC3126412

[B12] Nevin K. P. Woodard T. L. Franks A. E. Summers Z. M. Lovley D. R. (2010). Microbial electrosynthesis: feeding microbes electricity to convert carbon dioxide and water to multicarbon extracellular organic compounds. *mBio* 1 e00103–e00110 10.1128/mBio.00103-10 20714445 PMC2921159

[B13] Pant D. Singh A. Van Bogaert G. Gallego Y. A. Diels L. Vanbroekhoven K. (2011). An introduction to the life cycle assessment (LCA) of bioelectrochemical systems (BES) for sustainable energy and product generation: relevance and key aspects. *Renew. Sust. Energ. Rev.* 15 1305–1313 10.1016/j.rser.2010.10.005

[B14] Rittmann B. E. McCarty P. (2001). *Environmental Biotechnology: Principles and Applications.* New York: McGraw-Hill.

[B15] Rosenbaum M. Aulenta F. Villano M. Angenent L. T. (2011). Cathodes as electron donors for microbial metabolism: which extracellular electron transfer mechanisms are involved? *Bioresour. Technol.* 102 324–333 10.1016/j.biortech.2010.07.008 20688515

[B16] Rosenbaum M. E. Henrich A. W. (2014). Engineering microbial electrocatalysis for chemical and fuel production. *Curr. Opin. Biotechnol.* 29 93–98 10.1016/j.copbio.2014.03.003 24709348

[B17] Tremblay P.-L. Zhang T. Dar S. A. Leang C. Lovley D. R. (2013). The Rnf complex of *Clostridium ljungdahlii* is a proton-translocating ferredoxin:NAD+ oxidoreductase essential for autotrophic growth. *mBio* 4 e00406–e00412 10.1128/mBio.00406-12 23269825 PMC3531802

[B18] Ueki T. Nevin K. P. Woodard T. L. Lovley D. R. (2014). Converting carbon dioxide to butyrate with an engineered strain of *Clostridium ljungdahlii*. *mBio* 5 e01636–e01614 10.1128/mBio.01636-14 25336453 PMC4212834

[B19] Zhang T. Nie H. Bain T. S. Lu H. Cui M. Snoeyenbos-West O. L. (2013). Improved cathode materials for microbial electrosynthesis. *Energy Environ. Sci.* 6 217–224 10.1039/C2EE23350A

